# The Effects of Learning Transfer on Clinical Performances Among Medical Staff: A Systematic Review of Randomized Controlled Trials

**DOI:** 10.3389/fpubh.2022.874115

**Published:** 2022-07-05

**Authors:** Yung-Chieh Tung, Ying Xu, Yu-pei Yang, Tao-Hsin Tung

**Affiliations:** ^1^Division of Cardiovascular Surgery, Kaohsiung Veterans General Hospital, Kaohsiung, Taiwan; ^2^Institute for Hospital Management, Tsing Hua University, Shenzhen, China; ^3^Department of Hematology, Taizhou Hospital of Zhejiang Province Affiliated to Wenzhou Medical University, Linhai, China; ^4^Evidence-Based Medicine Center, Taizhou Hospital of Zhejiang Province Affiliated to Wenzhou Medical University, Linhai, China

**Keywords:** learning transfer, clinical performances, medical staff, systematic review, randomized controlled trials

## Abstract

**Purpose:**

This study aimed to evaluate the influence of learning transfer on the clinical performance of medical staff.

**Methods:**

We searched PubMed, Embase, and the Cochrane Library for all associated studies without any language restrictions from the inception until 31 December 2021.

**Results:**

This systematic review screened out 14 eligible studies that met the inclusion criteria. Most of these studies showed that learning transfer contributed to the clinical performance of medical staff. Through education, or when knowledge and skills have common basic principles, learning transfer will be more apparent than for those who learn by themselves and those without simulation training.

**Conclusions:**

The findings of this review support an association between learning transfer and the clinical performance of medical staff. However, it was noted that due to the lack of relevant research and the major differences in the methods and indicators used in previous studies, we are restricted in conducting an effective meta-analysis. Further comprehensive trials will be needed to assess the impact of learning transfer on the clinical performance of medical staff.

**Systematic Review Registration:**

PROSPERO, identifier: 341439.

## Introduction

It has been widely acknowledged that the system of healthcare is complicated. Hundreds of pieces of clinical data are generated from the healthcare process, such as the patient's history, examination findings, and investigation results. Correct diagnosis can be defined by analyzing these huge amounts of data accurately. With the rapid expansion of the knowledge base of diseases and their management, the complexity of the system of healthcare is inevitably aggravated (1). Clinical performance should meet the highest standards based on adequate knowledge, determination, technology, and attitudes at different levels of clinical practice (2). Medical professionals should be able to implement technical, intelligent, and elevated skilled clinical practices so as to offer reliable and high-quality medical care to each patient (3). In order to improve the clinical performance of medical staff, it is essential to arrange continuing education and to make a great effort to upgrade medical professionals' learning and skills through different educational courses.

For decades, researchers have examined the practical concept of learning transfer. Learning complicated abilities for individuals, according to Gagne (4), requires comprehension based on adequate knowledge, implying that learning is a cumulative process. Transfer of learning, according to Ellis (5), occurs when “experience or performance on one task has an effect on performance on a subsequent one.” McKeachie (6) defined transfer as “The application of earlier learning in a condition that is not the same as the learning situation.” Learning transfer was later described as the extent to which knowledge (simple or complicated), skills (conceptual, interpersonal, or technical; open or closed), and competencies acquired during training are transferred to the job (7–10). It is also noteworthy that learning transfer, on the other hand, is not a static concept, and its meaning varies depending on how it is defined and utilized before, during, and after the learning process. Learning transfer is mainly across test patterns, implantation and judgment matters, problems involving clinical diagnoses, and mediator and associated word suggestions (11). Transfer of learning is critical in education, as the context of learning varies with the context of the application (12). Medical staff are expected to build a framework of the cognitive foundation from books, lectures, or simulations, draw principles from their prior knowledge and experiences, and apply learning in their workplace, building their ability to manage and solve problems. In nursing, it is reported that the transfer of learning has led to the effectiveness of simulation and debriefing experiences (13–15).

From the clinical viewpoint, the better the learning transfer, the more challenging the appointed assignments could be, and the more active and creative the results. Although the main processes of learning transfer include formal learning activities (e.g., maintenance education or job training programs) and self-directed informal learning activities, a previous study reported low levels of learning transfer among members of an institution (3). In addition, a case-based blended learning (CBBL) framework which utilized the flexibilities of an e-learning platform has highlighted that E-Case-Based Learning is effective in promoting the outcome of performance and is an essential way of learning and discovering (16, 17).

It is valuable to explore the practical implications of the effectiveness of learning transfer used in medical education and related training circumstances. Whether training transfer is associated with clinical services is an essential question warranting investigation. Thus, we conducted this systematic review to further evaluate the influences of learning transfer on clinical performances among medical staff.

## Materials and Methods

### Literature Review

We performed this study in accordance with guidelines outlined in the Preferred Reporting Items for Systematic Reviews and Meta-Analyses (PRISMA) (PROSPERO ID: 341439). We conducted a comprehensive search for relevant studies (without language limitations) from major online databases, such as PubMed, Embase, and the Cochrane Library, from inception to 31 March 2022. Two independent reviewers scanned the literature and included the eligible studies by common consensus after multiple rounds of screening.

### Data Sources and Search Methods

The search process included (i) reading the reference section of all relevant research carefully; and (ii) manually searching abstracts of key journals and papers published at major annual conferences. The search terms used were a mix of (“learning transfer” [All Fields] OR “boundary crossing training” [All Fields]) AND (“clinical performance” [All Fields] OR “academic theoretical knowledge” [All Fields] OR “professional practice experience” [All Fields]). We also checked the reference lists of the screened studies to identify other similar studies. The search strategy is shown in Table 1. We included experimental studies that examined the influence or effectiveness of learning transfer on the clinical performance of medical staff. The PICOS criteria are used to select the eligible studies. Studies were included if they satisfied the following inclusion criteria: (1) The study was limited to RCTs and humans; (2) All participants are medical staff; (3) The study included both an experimental group and a control group. The experimental group was subjected to a learning lesson while the control group was performed without learning a lesson. (4) The study reported the effect of learning transfer on the performance of medical staff in each group. The exclusion criteria were as follows: (1) unqualified study design, such as non-RCT design, single-arm extension study, observational studies; (2) case reports, editorials, or reviews; (3) duplicated reports.

**Table 1:** Search strategy.

**Database**	**Searching keywords**
Cochrane library	(1) Learning transfer: 1510 (2) Boundary crossing training: 8 (3) Clinical performance: 79053 (4) Academic theoretical knowledge: 302 (5) Professional practice experience: 1607 (6) #1 OR #2: 1517 (7) #3 OR #4 OR #5: 79921 (8) #6 AND #7: 673
PubMed	(1) Learning transfer: 21039 (2) Boundary crossing training: 772 (3) Clinical performance: 1129461 (4) Academic theoretical knowledge: 894 (5) Professional practice experience: 28844 (6) #1 OR #2: 21800 (7) #3 OR #4 OR #5: 1156646 (8) #6 AND #7: 2116
Embase	(1) Learning transfer: 228 (2) Boundary crossing training: 0 (3) Clinical performance: 12913 (4) Academic theoretical knowledge: 1 (5) Professional practice experience: 11 (6) #1 OR #2: 228 (7) #3 OR #4 OR #5: 12925 (8) #6 AND #7: 2

### Data Extraction and Quality Assessment

For all articles included, we extracted the following information from the original articles: first author, publication year, country, database, study duration, study design, study subjects, mean age of study subjects, gender of the study subjects, and outcomes. Two reviewers independently performed an analysis of methodological quality. The quality assessment included the following items: allocation generation and concealment, blinding, follow-up duration, loss follow-up (%), and data-analysis method (intention-to-treat or per protocol). Divergences were resolved through discussion and consensus. Further, we used version 2 of the Cochrane risk-of-bias tool for randomized trials (RoB 2) to assess the risk of bias for Systematic Reviews of Interventions. Any disagreement was resolved through discussion with a third author. All analyses were performed by Review Manager version 5.4.1.

## Results

### Study Characteristics

**Figure 1:**
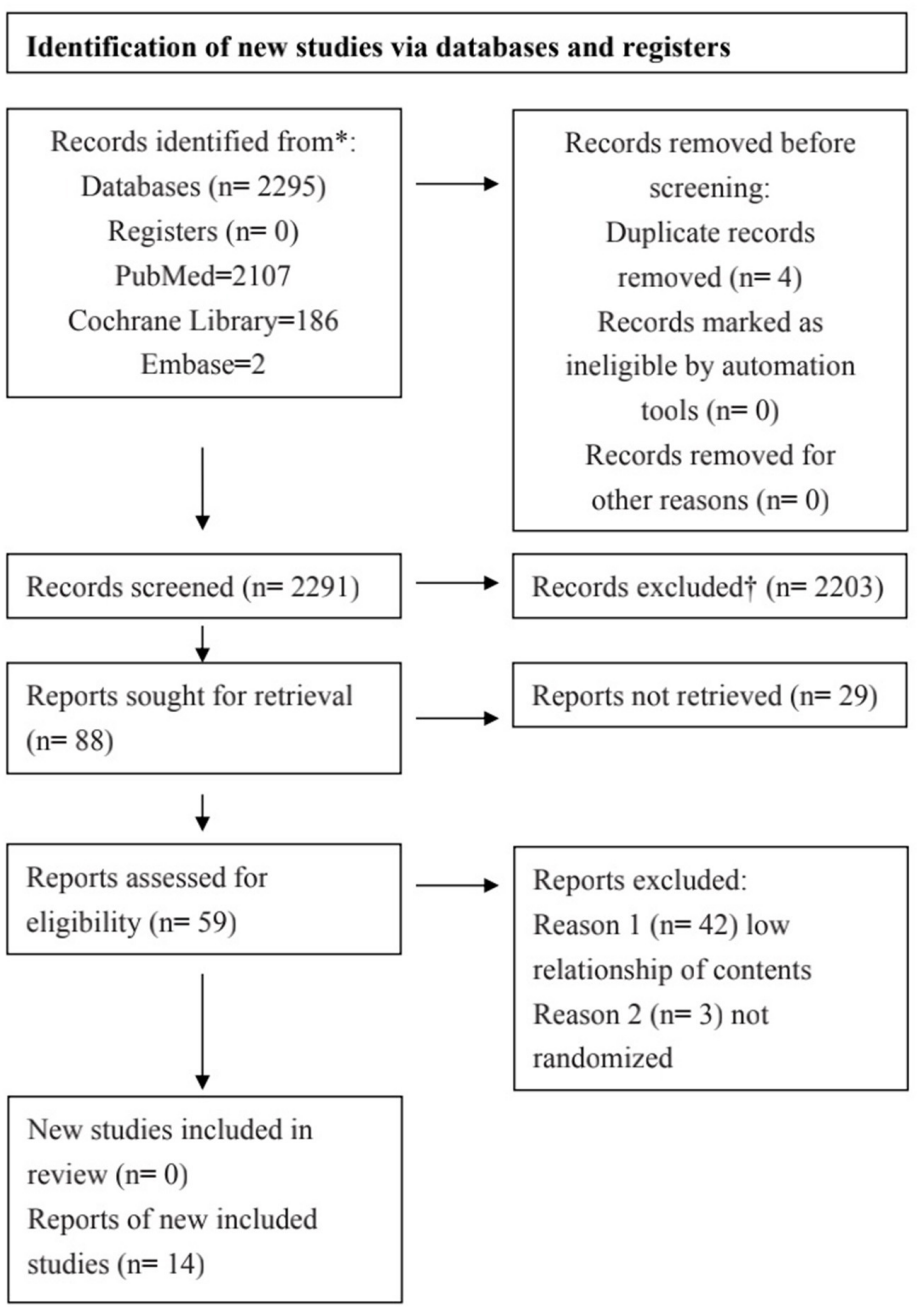
PRISMA (Preferred Reporting Items for Systematic Reviews and Meta-Analyses) flow diagram.

The results of the systematic review are presented in Figure 1. We identified a total of 14 studies related to the transfer of learning after a thorough review of all papers. The characteristics of the studies are listed in Table 2. Among the studies considered in this paper, seven were conducted in Europe, three were in Canada, two were in the United States, and one each was conducted in Australia and South Korea.

**Table 2:** Characteristics of randomized controlled trials.

**References**	**Year**	**Country**	**Inclusion criteria**	**Group**	**Intervention**	**Study subject**	**Mean age (years)**	**Gender (M/F)**	**Outcome measures**
Anastakis et al. (18)	1999	Canada	PGY-1	Text only, bench model training, or cadaver model training	A 3-day period training on six operative procedures using one of three methods and 1 week later exam	23 PGY-1 surgical residents	NA	NA	Both bench and cadaver training were superior to text learning and that bench and cadaver training were equivalent
Jensen et al. (19)	2005	Sweden	Volunteered to participate; One criterion for participating was willingness to verbalize.	The rabbits-and-foxes task and the reindeer-and-lichen task	A learning session with the first task before being tested in the second task and the other only performed the second task	28 undergraduate psychology students	22 (range 18-30)	0/28	A significant transfer effect from the rabbits and foxes task to the reindeer-and-lichen task
Heaven et al. (20)	2005	UK	25 (41%) were community based, 13 (21.3%) worked in a hospital, whilst 23 (37.7%) worked across both domains. The nurses in the study were relatively experienced.	Receive either communication skills training followed by clinical supervision or communication skills training alone	All attended a 3-day communication skills training workshop. Twenty-nine were then randomized to 4 weeks of clinical supervision	61 clinical nurse specialists	42 ± 7.4	1/60	Each nurse's communication skills with real patients were assessed a three time points. (1) Before training and supervision (baseline), (2) immediately after the supervision intervention (post) and (3) 3 months after the post intervention (follow-up). Only those who experienced supervision showed any evidence of transfer.
Butler et al. (21)	2013	USA	No prior experience performing arthroscopic surgery	Whether trained to perform diagnostic arthroscopy of the knee on anatomic dry models before trained only on cadaveric specimens.	All students were trained to perform diagnostic knee arthroscopy on cadaveric specimens. For the students in the experimental group, the cadaveric session took place between 8 and 21 days following their initial training with the dry models	14 medical students	NA	8/6	The mean number of trials to demonstrate minimum proficiency was significantly lower in the experimental group (2.57) than in the control group (4.57) (*p* < 0.01). The mean time to demonstrate proficiency was also significantly less in the experimental group (37.51 minutes) than in the control group (60.48 min) (*p* < 0.01).
Bjurström et al. (22)	2013	Denmark	The surgeons had different levels of experience with thoracoscopy. Inclusion criteria of the medical students consisted of having no previous experience with endoscopic surgery and having reached their third year or further along	Group 1: the surgeons, performed 2 consecutive attempts of the first 2 training tracks Group 2: performed the test directly without training Group 3: 3 h of independent training using the VT simulator Group 4: the educator-guided students received 3 h of guided, goal-oriented training	A control group and the group of surgeons were tested with no previous simulator training. A self-guided training group and an educator guided training group trained for 3 h on 3 scenarios of increasing fidelity and difficulty before taking a standardized test	10 surgeons and 30 medical students	30 (range 21–65)	30/10	The control group and the self-guided training group performed significantly worse than the experienced surgeons (*P* = 0.012 and *P* = 0.010, respectively)
Ferguson et al. (23)	2015	UK	Students rotating through their orthopedic attachment at the hospital and had no previous experience of arthroscopy	Knee or shoulder arthroscopic	After nine task repetitions over 3 weeks on one model, each participant undertook the simulation task of the other anatomical joint.	18 medical students	NA	NA	There was no immediate evidence of skill transfer, with a significant drop in performance between the final training episode and the transfer task (all parameters *p* < 0.003)
Tolsgaard et al. (24)	2015	Denmark	All participants had an equal knowledge base and minimal practical ultrasound experience and the inclusion criterion required participants to be <4 months from medical graduation	Single or dyad	A 2-h training programme on a transvaginal ultrasound simulator before the transfer test	Medical students	28 (range 23-34)	1/29	The dyad group demonstrated higher training efficiency in terms of simulator score per number of attempts compared with the single-student group (*p* = 0.03).
Rutherford-Hemming et al. (25)	2016	USA	Eligible nurses worked full- or part-time in an inpatient mother-baby unit (post partum) or birthing center	Simulation or online self-study module	Direct observation and completion of a standardized instrument by the observer at 3 time points, using a validated 12-item Neurologic Knowledge Assessment and a 14-item performance skill checklist.	Nurses	49.5 ± 10.5	NA	They had similar mean levels on Neurologic Knowledge Assessment scores in short-term (*P* = 0.86) and longterm (*P* = 0.59), but these mean scores were not significant
Kulasegaram et al. (26)	2017	Canada	Students were generally new to the anatomy and physiology concepts used in the experiment.	The Analogy and No Analogy; the one-, two- and three organ-system conditions.	Each participant learned three physiology concepts using a standard clinical explanation and diagram provided by an expert clinician (AN) or the standard explanation and an analogy illustrating deep structure, and 1- week delay to complete a new transfer test	90 first-year psychology students	NA	NA	The analogy condition had a smaller difference between near and far transfer performance (0.99 vs. 0.91) compared with the no-analogy group (1.21 vs. 0.77); average far transfer score was higher for the two- and three-organ-system groups compared to the one-organ-system group.
Kulasegaram et al. (26)	2017	Canada	Different from experiment 1	The Analogy and No Analogy; the one- and two- organ-system conditions.	Randomized again to practicing with one or two organ systems for laminar flow and Laplace's law, after completing learning, participants took a multiple-choice test to test recall and a similarity categorization test	40 first-year psychology students	NA	NA	There were no significant differences between any groups on MCQ testing
Yang et al. (27)	2018	Germany	Participants were laparoscopically naive medical students and showed a special interest in surgery	Group 1: An appendectomy training on the VRS before the tutorial procedural tasks of LC Group 2: the tutorial procedural tasks of LC directly	Whether training on the VRS before the tutorial procedural tasks of Laparoscopic cholecystectomy	medical students	24.5 (range 21-33)	12/32	Participants in group 1 needed significantly less movements (388.6 ± 98.6 vs. 446.4 ± 81.6; *P* < 0.05) as well as shorter path length (810.2 ± 159.5 vs. 945.5 ± 187.8 cm; *P* < 0.05)
Genç and Öner (28)	2019	Canada	Participants were excluded if they had previous LP training	Procedural Only, Integrated in Sequence, and Integrated for Causation	A self-regulated simulation-based LP training session and a follow-up session 1 week later	66 medical students	NA	NA	Participants receiving an integrated instructional video performed significantly better on transfer through their intervention's positive impact on conceptual knowledge (all *p* < 0.01)
Beattie et al. (29)	2020	Australia	All participants reported normal or corrected-to-normal vision, normal stereoacuity, and no prior laparoscopic experience (including no formalisedlaparoscopic skills training with a simulator, and no hands-on laparoscopic experience in an operational context, e.g., as a surgical assistant)	The 2D → 3D and 3D → 2D groups and he 2D → 2D and 3D → 3D groups	Proficiency-based training in six laparoscopic training tasks; testing included two further repetitions of all tasks under test conditions	60 medical students	24.78 ± 3.24(range 19–34)	32/28	The groups trained in 3D demonstrated superior training performance and took fewer repetitions to reach proficiency than the groups trained in 2D. The groups tested in 3D also demonstrated superior test performance compared to those tested in 2D
Anacleto et al. (30)	2021	Portugal	Participants with no previous experience with laparoscopy or laparoscopic exercises.	Group 1 watched the VMT in both trials and Group 2 watched, firstly, the original E-BLUS examination video and, in the second trial, the VMT.	Take five minutes to practice and get familiar with both tasks, after the exercises in the first trial, in both trials and groups, the first exercise to be performed was the PT followed by the NG.	42 final year medical students	NA	NA	After watching the VMT, a decrease in the total number of errors in PT and NG exercises was observed in the participants who previously watched the E-BLUS video (*p* = 0.001 and *p* = 0.002, respectively).
Lee and Son (31)	2021	South Korea	All participants had completed a pre-requisite maternity nursing course and had basic knowledge related to women's health nursing before the study.	One engaged in S-PBL based on Pap smear knowledge and the other participated in a Pap smear demonstration based on Pap smear knowledge	After the intervention, self-confidence, learner satisfaction, and critical thinking were evaluated, using a structured questionnaire to measure learning transfer related to Pap smears, both for the experimental and control group.	Third-year nursing students	22.31 ± 2.42	20/85	Two groups showed that the general characteristics, self-confidence (*t* = 0.51, *p* = 0.612), learner satisfaction (t = 0.72, *p* = 0.475), and critical thinking (t = 1.42, *p* = 0.158) were homogeneous (*p* > 0.05)

### Quality Assessment

Table 3 shows the results of a methodological quality assessment of all included studies. We considered inadequate allocation concealment and sequence generation the most common sources of potential bias. Due to the few studies included and the degree of heterogeneity observed in the study design, interventions, and outcome indices, meta-analysis was considered impractical.

**Table 3:** Methodological quality assessment of the included studies.

**References**	**Year**	**Allocation generation**	**Allocation concealment**	**Double blinding**	**Follow-up duration**	**Loss to follow-up**	**Data analysis**	**Other bias**
Anastakis et al. (18)	1999	PGY-1 surgical residents	Adequate	No	A 3-day period training and 1 week later exam	0	ITT	—
Jensen et al. (19)	2005	Undergraduate psychology students, all female	Adequate	No	2003~2005	0	ITT	—
Heavenet al. (20)	2005	Clinical nurse specialists	Adequate	No	3 months	0	ITT	—
Butler et al. (21)	2013	Medical students	Unclear	No	21 days	0	ITT	—
Bjurström et al. (22)	2013	10 surgeons and 30 medical students	Unclear	No	2 months	0	ITT	—
Ferguson et al. (23)	2015	Medical students	Adequate	No	One week	0	ITT	—
Tolsgaard et al. (24)	2015	Medical students	Adequate	No	6 months	20	PP	—
Rutherford-Hemming et al. (25)	2016	Nurses	Adequate	Single blind	2 months	3	PP	—
Kulasegaram et al. (26)	2017	First-year psychology students	Adequate	No	One week	17	PP	—
Yang et al. (27)	2018	Medical students	Unclear	Unclear	10 months	8	PP	—
Genç and Öner (28)	2019	Medical students	Adequate	Single blind	One week	0	ITT	—
Beattieet al. (29)	2020	Medical students	Unclear	Unclear	N/A	0	ITT	—
Anacleto et al. (30)	2021	Medical students	Adequate	No	2018.09	21	PP	—
Lee and Son (31)	2021	nursing students	Adequate	Single blind	2 months	0	ITT	—

**Figure 2:**
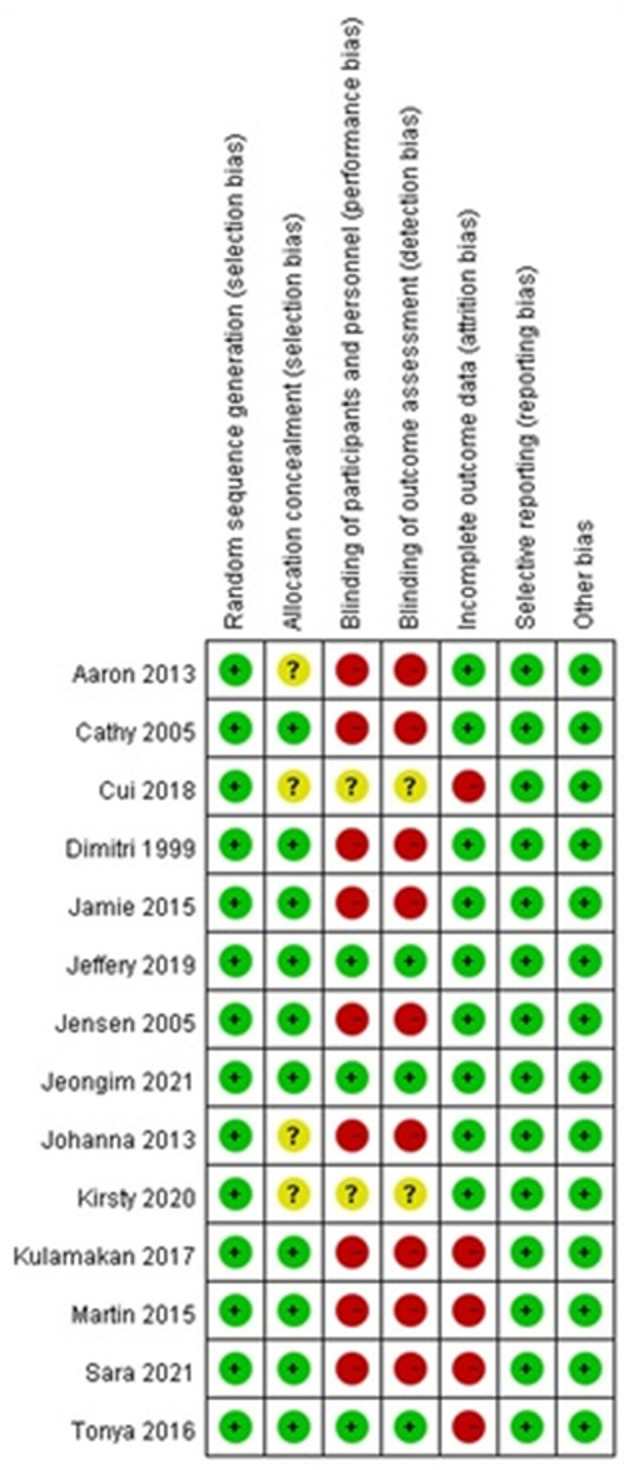
Risk of bias summary.

Figure 2 presents a summary assessment of bias risk. Butler et al. (21), Bjurström et al. (22), Yang et al. (27), and Beattie et al. (29) did not clearly describe how the research populations are selected. Yang et al. (27) and Beattie et al. (29) did not clearly explain whether the participants were blinded. Yang et al. (27), Tolsgaard et al. (24), Rutherford-Hemming et al. (25), Kulamakan et al. (26), and Anacleto et al. (30) lost a number of research objects to follow-up, and therefore we must assume a high risk of bias. Setting the issue of uncleared blinded participants aside, all but Rutherford-Hemming et al. (25), Genç and Öner (28), and Lee et al. (31) did not blind participants; thus, their assessment of outcomes must be regarded as questionable.

### Effective Learning Transfer in the Final Task

Jensen et al. (19) reported a significant transfer effect when performing the second task, as there was a learning session in the first task. Butler et al. (21) reported that medical students were trained to perform diagnostic arthroscopy of the knee on anatomic dry models before training on cadaver specimens. Their average number of minimum proficiency tests and the average time of proficiency were significantly less than for those who only trained on cadaveric specimens. Kulamakan et al. (26) performed two sequential experiments. In Experiment 1, increasing context variation and conceptual analogies both significantly led to higher performance for far transfer. Experiment 2 demonstrated that even though there was a superficial similarity to previous examples, learners' shifts to using structural characteristics to classify new problems were caused by such analogies and context variation.

Yang et al. (27) reported that the participants performed appendectomy training in the virtual reality simulator before the tutorial procedural tasks of laparoscopic cholecystectomy, and needed significantly fewer movements and shorter path lengths than those who started with the tutorial procedural tasks of laparoscopic cholecystectomy directly. Anacleto et al. (30), after watching the video-mentored tutorial (VMT), observed a decrease in the total number of errors in peg-transfer (PT) and needle-guidance (NG) exercises in the participants who had watched the European Basic Laparoscopic Urologic Skills (E-BLUS) video before. Compared with the group in which students participated in a conventional demonstration of a Papanicolaou smear, Lee et al. (31) reported that self-confidence, learner satisfaction, and critical thinking were significantly higher in the simulation problem-based learning (S-PBL) group. After nine task repetitions over 3 weeks on one model, Ferguson et al. (23) reported that when the participants performing the knee and shoulder tasks swapped models, there was no immediate evidence of skill transfer.

### The Most Effective Learning Transfer in Different Training Methods

Anastakis et al. (18) reported that bench and cadaver training were both superior to text learning and were equivalent. Tolsgaard et al. (24) reported that compared to the single-student composition, dyads illustrated higher training efficiency in terms of simulator score per number of attempts. Compared with nurses in the online self-study module, Rutherford-Hemming et al. (25) reported that the simulation group showed higher levels in both short-term and long-term skill performance.

Genç and Öner (28) reported that participants receiving an integrated instructional video performed significantly better on transfer than the procedural-only video. Beattie et al. (29) reported that groups trained in 3D and tested in 3D demonstrated superior training performance and took fewer repetitions to reach proficiency than the group in 2D.

### The Effect of Guidance on Learning Transfer

The last two papers addressed in this study discuss the relationship between teacher guidance and learning transfer. While conducting research with 61 clinical nurse specialists as participants, Heaven et al. (20) assessed the three time points and observed that only those who experienced supervision showed any demonstration of transfer. Although neither group promoted more revelation of clues or concerns, people in the experimental group responded more effectively to the revealing clues. Bjurström et al. (22) reported that experienced surgeons performed significantly better than the control group and the self-guided training group. Between the educator-guided training group and the experienced surgeons, there was no significant difference. Nevertheless, having an educator present during training seemed to have a beneficial effect.

## Discussion

### Clinical Implication

Our study integrated the current findings of 14 studies and illustrated the correlation between the learning transfer and clinical performance of medical staff. However, the experimental items in each article based on learning transfer are not all the same. Therefore, we cannot compare them to determine which methods of learning transfer ensure significant clinical performance for medical staff. In short, we can only understand the transfer of learning in different fields based on a synthesis of current findings.

Previous research showed that learning transfer is considered a major influence on clinical performance (3). Learning in hospital settings encompassed both formal and informal activities. Formal learning means formalized and standardized education, including career staff training, preceptorships, maintenance education, and job training. Nonetheless, owing to the nature of shift work and the organizational complexity and diversity, it is not possible to make sure that medical staff can improve their clinical performance only through formal learning. Informal learning, which consists of communication, interaction with others, role modeling, and team-based learning, is more flexible and plays an important role in developing the medical staff's clinical performance, especially professional practice experience (32, 33). Informal learning is not simply passive inputting of information but involves constructing the meaning of information actively by recording accumulated long-term memory and existing experience. Therefore, it is crucial to decide which approaches should be used in informal learning, supporting continual self-directed learning.

In this study, we found better task performance after medical students were trained in the virtual simulator, which indicates that when knowledge and skills had common basic principles, the learning transfer would be apparent (27). Learners apply the acquired knowledge to the new learning or work, illustrating the importance of learning transfer to the clinical performance of medical staff and cultivating their practical ability and creative spirit. With an effective transfer, learners can learn faster and better in a limited time and transfer more accurately in the appropriate environment. It seems that learning transfer also depends on the direct instruction of teachers (3, 20), as instruction can more effectively promote learning transfer. The report showed no significant difference between the training group guided by educators and experienced surgeons (22). Nevertheless, strengthening guidance during learning can help students to improve their specific knowledge to general principles as early as possible.

The systematic review found that despite the lack of consistency in the duration of the intervention, practice time, assessment, and outcome measure, there is a significant learning transfer in the final task after interventions such as different training methods and guidance. According to the results, in order to make an effective learning transfer, professionals should focus on the following three points: (1) Look for the similarities between concepts and principles; (2) notice the summary of learning methods, that is, master the method of solving problems in the learning process, and (3) accumulate a wide range of learning experience in all aspects.

### Clinical Practice

Owing to the developments of new technologies and the shift in the medical paradigm, E-Learning within the CBBL framework is seen as a very promising tool to prompt the advancement of learning transfer. Turk et al. explored that E-Learning within CBBL framework not only facilitates the creation of up-to-date teaching content but also addresses difficulties in transforming declarative knowledge into procedural knowledge and skill (34). Lütgendorf-Caucig highlighted that the integration of different teaching modalities is beneficial for the knowledge acquisition for clinical decision making in a multidisciplinary environment like oncology (35). It has been also emphasized in other studies that CBBL is an effective way in gaining improvements in performance among medical staff and is essential for associative and procedural learning that is necessary for clinical reasoning processes (36–39). Hence, we would recommend that the concept of interactive CBBL methods should be developed further and applied in other medical fields. To guarantee the high quality and employ correct didactic dimensions in terms of constructing the interactive questions, it may be helpful to create a guideline for question generation with the collaboration of medical education experts from their research field. Meanwhile, in order to provide a diversity of CBBL materials, further scientific, methodological, theoretical, and practice-based breakthroughs must be achieved.

### Strength and Limitation

We individually evaluated these studies using assessment tools and covered most of the articles related to learning transfer. However, several limitations need to be addressed. First, few studies included questions on the reliability of the research results and the strength of the conclusions. Second, due to the considerable heterogeneity of research design and outcome variables, it was impossible to perform an effective meta-analysis. Third, the research objects participating in our review may have differed in analysis and generalizability. Finally, there may be interference from other related factors. Although the participants in some studies are similar in age, their sex ratios are quite different; moreover, nearly half of the studies did not clearly indicate this. In addition, there is currently a lack of studies that provide the quantitative results to meet the condition of performing an effective meta-analysis. We recommend that researchers conduct randomized controlled trials to further evaluate this correlation. We also recommend a study comparing the transfers of different interventions to provide more comprehensive and general findings.

## Conclusion

Current evidence supports an association between learning transfer and the clinical performance of medical staff. However, it was noted that due to the lack of relevant research and the large differences in the methods and indicators used in previous studies, we were unable to conduct an effective meta-analysis. To summarize, medical staff should learn the importance of learning transfer and reinforce this ability through interdisciplinary teamwork and communication. Multi-disciplinary teaching approaches, assessments of existing systems and frameworks, and continuous technical improvements are still warranted in the future to optimize the current method of learning transfer and help medical staff make effective clinical decisions, as well as guarantee persistent satisfaction.

## Data Availability Statement

The raw data supporting the conclusions of this article will be made available by the authors, without undue reservation.

## Author Contributions

Y-CT: conception and design. Y-CT, YX, and Y-pY: acquisition, analysis or interpretation of data, and statistical analysis. Y-CT and YX: drafting of the manuscript. T-HT: supervision. All authors have read and agreed to the published version of the manuscript.

## Conflict of Interest

The authors declare that the research was conducted in the absence of any commercial or financial relationships that could be construed as a potential conflict of interest.

## Publisher's Note

All claims expressed in this article are solely those of the authors and do not necessarily represent those of their affiliated organizations, or those of the publisher, the editors and the reviewers. Any product that may be evaluated in this article, or claim that may be made by its manufacturer, is not guaranteed or endorsed by the publisher.
